# Supplementary figures and images for: Adsorption Capacity of Silica SBA-15 and Titanosilicate ETS-10 toward Indium Ions
Source: Materials (Basel). 2023 Apr 18;16(8):3201. doi: 10.3390/ma16083201 (PMC10144871; doi:10.3390/ma16083201)

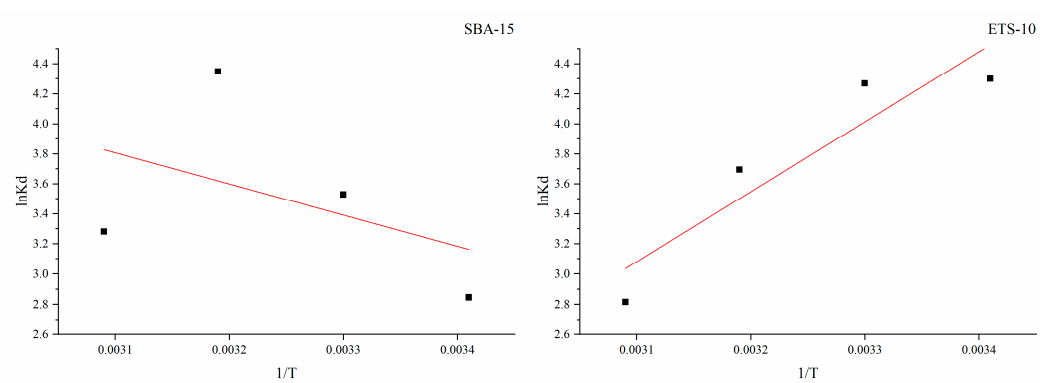

**Figure S1.** Plot of  $\ln K_d$  versus  $1/T$  for silica SBA-15 and titanosilicate ETS-10.

Supplement: Supplementary file 1 [file materials-16-03201-s001.zip › materials-2314745-SI.pdf]
